# Organic Phase‐Change Memory Transistor Based on an Organic Semiconductor with Reversible Molecular Conformation Transition

**DOI:** 10.1002/advs.202205694

**Published:** 2022-12-03

**Authors:** Yongxu Hu, Lei Zheng, Jie Li, Yinan Huang, Zhongwu Wang, Xueying Lu, Li Yu, Shuguang Wang, Yajing Sun, Shuaishuai Ding, Deyang Ji, Yong Lei, Xiaosong Chen, Liqiang Li, Wenping Hu

**Affiliations:** ^1^ Tianjin Key Laboratory of Molecular Optoelectronic Sciences Department of Chemistry Institute of Molecular Aggregation Science Tianjin University Tianjin 300072 China; ^2^ Shenzhen Key Laboratory of Polymer Science and Technology College of Materials Science and Engineering College of Physics and Optoeletronic Engineering Shenzhen University Shenzhen 518060 China; ^3^ Haihe Laboratory of Sustainable Chemical Transformations Tianjin 300192 China; ^4^ Fachgebiet Angewandte Nanophysik Institut für Physik & IMN MacroNano Technische Universität Ilmenau 98693 Ilmenau Germany; ^5^ Joint School of National University of Singapore and Tianjin University International Campus of Tianjin University Fuzhou 350207 China

**Keywords:** molecular conformation, molecular devices, organic electronics, organic semiconductor, phase‐change memory

## Abstract

Phase‐change semiconductor is one of the best candidates for designing nonvolatile memory, but it has never been realized in organic semiconductors until now. Here, a phase‐changeable and high‐mobility organic semiconductor (3,6‐DATT) is first synthesized. Benefiting from the introduction of electrostatic hydrogen bond (S···H), the molecular conformation of 3,6‐DATT crystals can be reversibly modulated by the electric field and ultraviolet irradiation. Through experimental and theoretical verification, the tiny difference in molecular conformation leads to crystalline polymorphisms and dramatically distinct charge transport properties, based on which a high‐performance organic phase‐change memory transistor (OPCMT) is constructed. The OPCMT exhibits a quick programming/erasing rate (about 3 s), long retention time (more than 2 h), and large memory window (i.e., large threshold voltage shift over 30 V). This work presents a new molecule design concept for organic semiconductors with reversible molecular conformation transition and opens a novel avenue for memory devices and other functional applications.

## Graphical abstract

Organic phase‐change semiconductor is one of the most promising candidates to design nonvolatile memories, which is not realized until now. This work first reports a novel principle to reversibly change the crystal phase of an organic semiconductor by modulating the molecular conformation transition, based on which high‐performance organic phase‐change memory transistors are constructed.

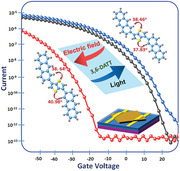

## 1 Introduction

With the advent of the internet of things, artificial intelligence networks, and big data era, the explosive growth of information data produces a huge need for the massive storage of data.^[^ ^1^, ^2^, ^3^, ^4^, ^5^, ^6^, ^7^, ^8^, ^9^ ^]^ Among various emerging memories, phase‐change memory (PCM), a widely used technology for constructing high‐performance nonvolatile memory, has attracted much attention by virtue of its long retention time, low power consumption, fast access speed, and high reliability.^[^ ^10^, ^11^, ^12^, ^13^, ^14^ ^]^ The crystal phases of traditional inorganic phase‐change materials, chalcogenide alloy semiconductors such as Ge_2_Sb_2_Te_5_, can be modulated by controlling temperature and exhibit dramatically distinct photoelectronic properties, which correspond to the different storage states.^[^ ^15^, ^16^, ^17^ ^]^ The combination of phase‐change and semiconductor characteristics endows PCMs with high potential for further realizing processing‐in‐memory, accurate charge modulation, and multilevel storage.^[^ ^18^, ^19^, ^20^ ^]^

With the increasing need for developing next‐generation flexible storage technologies, organic semiconductors become one of the most promising candidates for designing high‐performance and flexible PCMs.^[^ ^21^ ^]^ However, organic phase‐change semiconductors have never been reported. Different from the strong covalent bond of inorganic semiconductors, the weak intermolecular *π*–*π* interaction of organic semiconductors makes it easy to form irreversible phase change under the external stimulus, which causes high difficulty to reversibly change crystal phases in organic semiconductors.^[^ ^22^ ^]^ Therefore, developing an effective strategy to reversibly modulate the crystal phase of organic semiconductors is highly challenging and critical to construct organic phase‐change memory transistors (OPCMTs).

**Figure 1:**
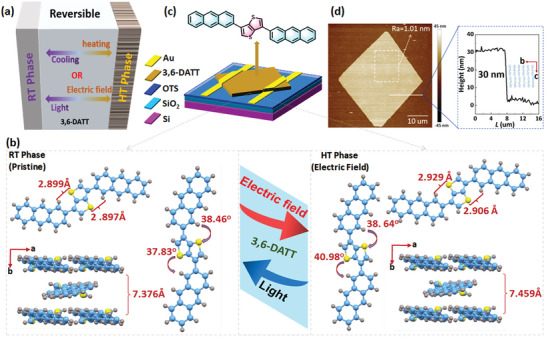
a) Schematic diagram of the reversible molecular conformation‐indued phase change under the external stimuli of heating/cooling or electric field/light irradiation. b) The crystal structures and molecular conformations of 3,6‐DATT in the RT phase and the HT phase. c) Device geometry of OPCMT based on 3,6‐DATT OFET. d) Atomic force microscope (AFM) image of 3,6‐DATT crystal and step heights of terrace layer as measured.

In this work, we developed a novel principle to change crystal phases of organic semiconductors by reversibly modulating the transition, based on which an OPCMT was constructed. Taking advantage of the twistable nature of the intramolecular electrostatic hydrogen bonding (S···H), we synthesized a p‐type organic semiconductor 3,6‐di(anthracen‐2‐yl)thieno [3,2‐b]thiophene (3,6‐DATT), whose torsion angle between their two aromatic planes can be reversibly modulated under electric field and ultraviolet irradiation (**Figure** **1**a,b). The molecular conformation transition induces crystalline polymorphisms including a high‐temperature (HT) phase and a room‐temperature (RT) phase, which exhibit dramatically distinct charge transport properties. The tiny molecular conformation transition guarantees the reversibility of phase‐change and electric properties. Furthermore, a high‐performance OPCMT was first constructed based on 3,6‐DATT (Figure 1c), which exhibits a fast programming/erasing rate (about 3 s), long retention time (more than 2 h), and large memory window (i.e., large threshold voltage shift over 30 V). This work opens up a new avenue for designing OPCMTs.

## 2 Results and Discussion

The organic semiconductor 3,6‐DATT was designed based on the following considerations: i) theoretical simulations predict that the introduction of electrostatic hydrogen bond (S···H) endows 3,6‐DATT with the potential for molecular conformation change under the external electric field;^[^ ^23^, ^24^, ^25^, ^26^ ^]^ ii) thieno[3,2‐b]thiophene has smaller reorganization energy compared with polycyclic aromatic hydrocarbons with the similar conjugation length, and the anthracene maintains the J‐aggregated mode, efficient charge transport characteristic, and excellent luminous efficiency.^[^ ^27^, ^28^, ^29^, ^30^ ^]^ As shown in Scheme S1 (Supporting Information), 3,6‐DATT was synthesized through one simple step of Suzuki coupling with a total yield of 44.5%. The molecular structure of 3,6‐DATT was determined by NMR spectroscopy, mass spectroscopy, elemental analysis, and X‐ray crystallographic analysis. The detailed description of molecular synthesis and characterization were shown in Section S1 (Supporting Information) and vide infra. To further analyze the crystal structures and exclude the possible influence of structural defects (i.e., grain boundaries and orientation disordering) on memory characterization in the following section, 3,6‐DATT single crystals were deposited on silicon wafers by physical vapor transport (PVT). The high thermal stability of 3,6‐DATT effectively guarantees the high growth quality of organic single crystal in the PVT process (Figure S1, Supporting Information). As shown in Figure 1d, the obtained microcrystals have an average thickness of 30 ± 1 nm and an ultraflat crystal surface (*R*_a_ = 1.01 nm). Meanwhile, the color of the obtained crystals changed when the sample was rotated under a cross‐polarized optical microscope (Figure S2, Supporting Information), which indicates the high‐ordered molecular packing and long‐range structural alignment.^[^ ^31^ ^]^

To clarify the pattern of conformation change in the intramolecular chemical bond of 3,6‐DATT in response to external stimuli, variable temperature X‐ray crystallographic measurements were conducted on 3,6‐DATT crystals. The 3,6‐DATT crystals exhibit two phases at room temperature (RT) and 120 °C, which are defined as the high‐temperature (HT) phase and the room‐temperature (RT) phase here, respectively. Interestingly, the HT and RT phases were tested in the same crystal by heating and cooling multiple times, and the phase change is fully reversible by varying temperatures. As shown in Figure 1b, Tables S1 and S2 (Supporting Information), the crystal structures of these two phases exhibited almost identical packing arrangements but only a tiny difference in torsion angle between the two aromatic planes. The torsion angle between anthracene and thieno[3,2‐b]thiophene at one side increased from 37.83° for the RT phase to 40.98° for the HT phases, and the S···H bond distance changed from 2.899 Å for the RT phase to 2.906 Å for the HT phase, resulting in the change of spatial configuration. This result experimentally demonstrated that the introduction of electrostatic hydrogen bonds (S···H) can effectively provide a path to control the molecular conformation under external stimuli, which is well consistent with our theoretical prediction.^[^ ^32^ ^]^ Remarkably, benefiting from this slight molecular conformation change with a tiny torsion angle, these two phases can be switched reversibly through the heating and cooling treatment. To date, such reversible molecular conformation transition has never been realized in other organic semiconductors crystal.

**Figure 2:**
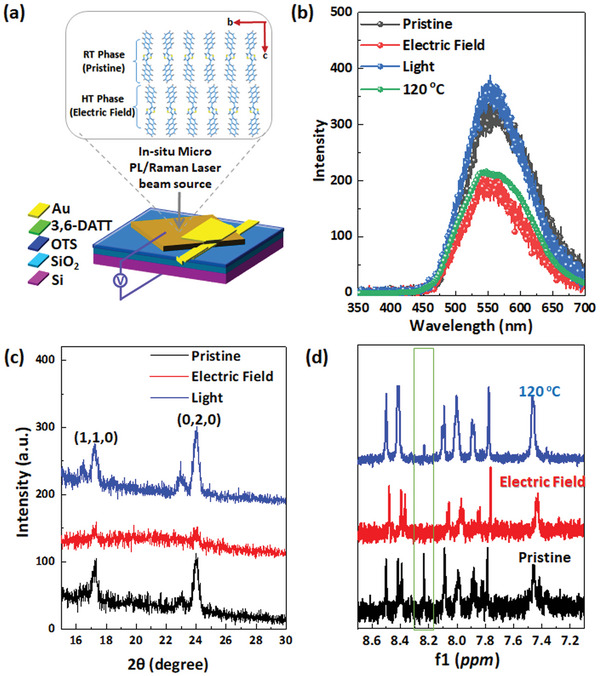
a) Schematic diagram of in‐situ micro‐photoluminescence/Raman measurement on 3,6‐DATT crystals, The inset shows the structures of the RT phase and the HT phase; b) In‐situ micro‐photoluminescence spectra of a 3,6‐DATT crystal during the molecular conformation transition. Pristine: as‐prepared crystals; Program: *V*_G_ = −21 V, *t* = 10 s; Erase: 365 nm UV illumination. c) In‐plane XRD patterns of 3,6‐DATT crystal before and after external stimuli. d) ^1^H NMR spectra of 3,6‐DATT at different states.

Although temperature modulation is widely used in inorganic PCMs, it is less suitable for OPCMTs due to the relatively weak thermal stability of organic components in organic optoelectronic circuits. To improve the compatibility of phase‐change modulation technology in organic memory, it is necessary to further develop milder stimuli for reversibly modulating molecular conformation transition. According to the theoretical prediction, the introduction of an electrostatic hydrogen bond (S···H) has the potential to control the molecular conformation under the external electric field. Through the verification by in‐situ micro‐photoluminescence and micro‐Raman spectroscopy, X‐ray diffraction (XRD) measurements, and nuclear magnetic resonance spectroscopy (NMR) measurements, the transition from the RT phase to the HT phase can be driven by the electric field, and the reverse process can be driven by ultraviolet light (365 nm) irradiation (Figure 1a,b). It is reported that the luminous properties of organic materials closely relate to the molecular packing state.^[^ ^33^ ^]^ As shown in **Figure** **2**a, we tested the photoluminescent (PL) properties of 3,6‐DATT crystal under the electric field by micro‐photoluminescence spectroscopy. As expected, the PL intensity of 3,6‐DATT crystals under the electric field is similar to that of the HT phase and is lower than that of the RT phase (Figure 2b). This result indicates that the molecular packing state of 3,6‐DATT after electric field treatment is similar to the HT phase. After being irradiated by 365 nm ultraviolet light, the quickly recovered PL intensity indicates the transition from the HT phase to the RT phase, which may derive from the photoinduced molecular motion effect.^[^ ^34^ ^]^ On the other hand, the in‐situ Raman measurements also confirmed the reversible molecular conformation change (for details, see Figure S4, Supporting Information).

It should be noted that the molecular conformation transition is a dynamic process. The HT phase and the RT phase always coexist with different ratios. As shown in Figure 1b and Figure S2 (Supporting Information), the as‐prepared single crystal (i.e., RT phase) has a long‐range structural alignment, while the degree of long‐range order will decrease with the transition to the HT phase. To verify this process, the in‐plane XRD patterns of 3,6‐DATT crystals were measured after electric field treatment (Figure 2c). The diffraction peaks of (110) and (020) planes become weaker compared with those of the RT phase because of the decreased degree of long‐range order. Meanwhile, ultraviolet light irradiation restores the diffraction intensity to its original state (i.e., RT phase). According to the theoretical prediction, the tiny torsion angle change derives from the S···H bond; therefore, the chemical environment of the H atom should change with the molecular conformation transition. ^1^H NMR spectra of 3,6‐DATT at high temperature and after electric field treatment were measured, which are similar to, but different from that at room temperature. The detailed temperature‐dependence ^1^H NMR spectra and the conformation transition process of the 3,6‐DATT were exhibited in Figure S5 (Supporting Information). As shown in Figure 2d, the change of torsion angle between anthracene and thieno[3,2‐b]thiophene modifies the chemical environment of the H atoms at the special positions and leads to the shift and evolution of NMR peaks (Figure S5, Supporting Information). These results directly prove the conformation transition and phase‐change process.

**Figure 3:**
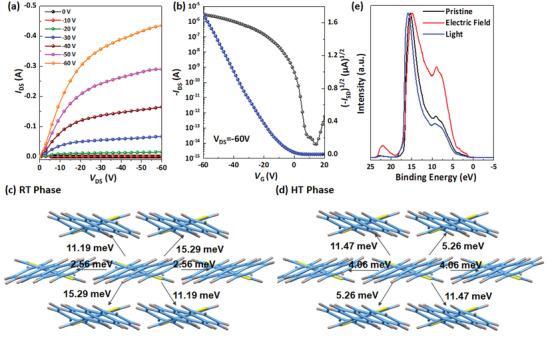
a) The output and b) transfer curve of OFETs with 3,6‐DATT crystals. Transfer integral of 3,6‐DATT crystals at: c) RT phase and d) HT phase. e) UPS energy distribution curve of 3,6‐DATT at different states.

Phase‐change‐induced conductance difference is the basic working mechanism of PCMs. To investigate the intrinsic charge transport properties of 3,6‐DATT crystals with HT and RT phases, microscale crystals were grown by the PVT method on octadecyltrichlorosilane (OTS)‐modified SiO_2_/Si wafers. Then, bottom‐gate top‐contact organic field‐effect transistors (OFETs) based on 3,6‐DATT crystals were manufactured (Figure 1b).^[^ ^35^, ^36^ ^]^ Representative output and transfer curves of 3,6‐DATT OFET with RT phase were presented in **Figure** **3**a,b. The OFETs based on 3,6‐DATT crystal exhibit an average mobility of about 1.0 ± 0.2 cm^2^ V^−1^ s^−1^ (from ten devices), high on/off ratio up to 10^7^, and a threshold voltage (*V*_T_) around −10 V. However, the mobility of 3,6‐DATT OFET with HT phase is only 0.04 ± 0.02 cm^2^ V^−1^ s^−1^. The dramatically distinct charge transport properties of 3,6‐DATT crystals with RT and HT phases guarantee their potential applications in PCM.

To understand more about the frontier molecular orbitals distribution of 3,6‐DATT in various molecular conformations that are closely related to the charge transport property, the density functional theory (DFT) calculations were further performed at B3LYP/6‐31G (d, p) level (Figure S6, Supporting Information). Both HOMO and LUMO of the RT phase and the HT phase of 3,6‐DATT crystals were well delocalized, which benefit superb optoelectronic properties. We used the Marcus theory to simulate the carrier transport process, and this model can be expressed as follows:

(1) $$k_{\mathrm{ij}}=V_{\mathrm{ij}}^{2}/h\sqrt{\left(\pi /\lambda k_{B}T\right)}\exp\left[{\left(\lambda +\Delta G_{\mathrm{ij}}^{0}\right)}^{2}/4\lambda k_{B}T\right]$$

*V*ij and *λ* are the transfer integral and reorganization energy, respectively, and $\Delta G_{\mathrm{ij}}^{0}$ is the Gibbs free energy difference.^[^ ^32^, ^37^ ^]^ We simulated the transfer integrals and reorganization energy of the two phases and found that the RT phase had a larger transfer integral than the HT phase (Figure 3c,d), indicating that the RT phase has a larger orbital overlap and better charge transport performance. However, in the terms of reorganization energy, there was no apparent difference between the two crystal phases, which further indicated that the strength of electron–phonon coupling to the perpendicular electric field did not change much (Figure S8, Supporting Information). The quantum nuclear tunneling model was used to investigate the charge transport properties.^[^ ^38^, ^39^, ^40^ ^]^ The mobility as well as the corresponding parameters were calculated by MOMAP.^[^ ^41^ ^]^ As a result, the theoretical mobilities of the RT phase and the HT phase are 4.51 and 2.25 cm^2^ V^−1^ s^−1^, respectively.

In addition to the charge transport properties, ultraviolet photoelectron spectrometer (UPS) measurements were performed to characterize the energy level, which is also a key factor‐influencing electronic applications. As shown in Figure 3e, the energy levels of the highest occupied molecular orbital (HOMO) were calculated to be 5.04 eV for the RT phase, 4.86 eV for the HT phase after electric field treatment, and 5.06 eV for the RT phase after UV light irradiation, respectively, which implied that RT phase of 3,6‐DATT crystal was beneficial to match with Femi level (5.0–5.2 eV) of the gold electrode, and also confirmed the reversible phase change by the electric field and UV light irradiation. It is noted that the large Schottky barrier of the gold/semiconductor contact region in the device with the HT phase causes an underestimation of mobility because more voltage is distributed to the contact region. As a result, the mobility difference between the theoretical value and experimental value in the device with HT phase is much larger than that in the device with RT phase.

**Figure 4:**
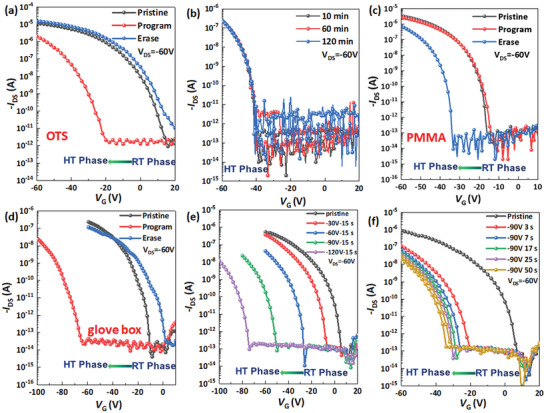
a) Transfer curves of the OPCMT device after programming (*V*_G_ = −90 V for 3 s) and erasing (365 nm light illumination for 3 s) operation. b) Retention time test of the OPCMT, reading at *V*_G_ = −90 V. c) Transfer curves of the 3,6‐DATT OPCMT on the PMMA‐treated SiO_2_/Si substrate. Programming: *V*_G_ = −90 V for 3 s. Erasing: 365 nm UV light illumination for 2 s. d) Transfer curves of the 3,6‐DATT OPCMT test in the glove box under the same test conditions as (a). Transfer curves of the 3,6‐DATT OPCMT under different programming conditions: e) different gate voltages for 15 s and f) fixed gate voltage of −90 V for different times.

The above results first reported an organic phase‐change semiconductor and effectively clarified the mechanism of phase‐change and modulation conditions, all of which solidly support the high feasibility to realize OPCMTs. Based on the reversible molecular conformation transition induced by the electric field and ultraviolet irradiation, a prototype OPCMT device was constructed with a field‐effect transistor configuration. **Figure** **4**a showed the transfer curves of the OPCMT device on OTS‐modified silicon wafers. The electrical characteristics under darkness at room temperature were set as a pristine state. After the programming operation (applying a gate voltage, *V*_G_, of −90 V for 3 s under darkness), the transfer curve shifted negatively with the threshold voltage (*V*_th_) shift from 0 to −32 V, which was set as the programming state. Once light irradiation (365 nm light irradiation of 262 µW cm^−2^ for 3 s) was applied, the transfer curve rapidly recovered to the pristine state, which was set as the erasing state. These results indicate that the molecular conformation transition had a fast response (3 s) and recovery (3 s) rate. Furthermore, the devices exhibited a long retention time of more than 2 h (Figure 4b) and good cyclability (Figure S11, Supporting Information), which indicated that the 3,6‐DATT can be maintained for a long time in the HT phase and would be beneficial for nonvolatile OPCMT applications.

To confirm that the above memory property stems from phase change rather than other factors, a series of comparative experiments were performed. It is known that the dielectric layer plays an important role in the shift of threshold voltage by charge trapping effect under an external electric field. Hence, 3,6‐DATT OFETs with polymethyl methacrylate (PMMA) dielectric and bare SiO_2_ dielectric were constructed as reference devices. As shown in Figure 4c and Figure S9 (Supporting Information), the same memory function as OPCMT was obtained. In the meantime, the possible influence of the air environment on 3,6‐DATT OPCMT performance was also excluded by measuring the memory in the glove box under the same test conditions (Figure 4d). To further optimize the programming conditions, we continue to explore the shift window of threshold voltage by systematically changing gate voltages and bias time (Figure 4e,f). The experimental results showed that −90 V and 3 s were the optimal programming conditions. Furthermore, we selected a reference molecule 2,5‐DAN without the S···H bond to construct OFET and test its memory property (Figure S10, Supporting Information), no similar memory phenomenon was observed, which further illustrated the unique properties of the S···H bond of molecule to reversibly modulate molecular conformation transition and realize OPCMT.

Finally, three points about this work are noted: i) the erasing process could not be realized under a positive electric field (e.g., *V*_G_ = 90 V, shown in Figure S12, Supporting Information). ii) it is demonstrated that the photoelectric response effect of organic semiconductors plays a minor effect in the erasing process (Figure S13, Supporting Information). iii) Compared with other memory transistors such as floating gate memory,^[^ ^42^, ^43^ ^]^ electret memory,^[^ ^44^, ^45^ ^]^ optoelectronic memory,^[^ ^46^, ^47^ ^]^ and ferroelectric memory,^[^ ^48^, ^49^ ^]^ the OPCMT in this work does not need to store charges in devices. The intrinsic conductivity difference of the two crystal phases can greatly decrease power consumption and be beneficial to device integration.

## 3 Conclusion

In summary, we first reported an OPCMT by reversibly modulating the molecular conformation of organic semiconductors. Benefiting from the intramolecular S···H bond and the tiny torsion angle between anthracene and thieno[3,2‐b]thiophene, the molecular conformation and crystal phase (i.e., RT phase and HT phase) of 3,6‐DATT crystals can be precisely and reversibly modulated by the electric field and ultraviolet irradiation. Interestingly, the tiny conformation difference produces dramatically distinct charge transport properties between these two phases. Based on this unique property of 3,6‐DATT, the OPCMT exhibits high performance including fast programming/erasing (about 3 s rate), long retention time (more than 2 h), and large memory window (over 30 V). Such memory mechanism by reversible molecular conformation transition has never been reported previously. These results demonstrated that the tiny and reversible molecular motion (torsion or vibration) would be a novel and effective way to design functional OSCs with tunable properties, which are highly promising for constructing memory devices and other functional applications. This work sheds new light on wide studies on molecular motion control and related device applications, such as data storage, sensing, and switch.

## 4 Experimental Section

### Materials and Instruments

All the starting materials and reagents were commercially available. Mass spectrometry (MS) was undertaken on a Bruker ultrafleXtreme MALDI‐TOF. ^1^H NMR and ^13^C NMR spectra were obtained in a JNM‐ECZ600R/S1 spectrometer. Elemental analysis was acquired by Vario Micro cube analyzer. UV–vis spectra were obtained on SHIMADZU UV‐2700 spectrophotometer. Photoluminescence (PL) spectra, lifetimes, and fluorescence quantum yields were recorded on a FLS1000 fluorescence. Thermo gravimetric analysis (TGA) was completed by a TG 209F3 thermal analyzer (Netzsch) analyzer at a heating rate of 10 °C min^−1^ from 50 to 720 °C under the nitrogen atmosphere. Ultraviolet photoelectron spectrometer (UPS) measurements were completed KRATOS Axis Ultra DLD spectrometer with He I (*h* = 21.22 eV) as the excitation source. AFM images were carried out on Digital Instruments Nanoscope III atomic force microscope in air. Thin single crystals X‐Ray diffraction was measured in Rigaku D/max‐2500 X‐ray diffractometer. The theoretical calculation of 3,6‐DATT was performed with GAUSSIAN‐09 adopting density functional theory (DFT)/B_3_LYP methods. Microphotoluminescence spectroscopy and micro‐Raman measurements were recorded on RENISHAW in Via reflex. X‐ray diffraction intensity data were collected at RT and 395 K on a Rigaku Saturn724 CCD diffract meter.

### Device Fabrication and Characterization

The single crystal field‐effect transistors (SC‐FETs) of 3,6‐DATT were fabricated on bare SiO_2_/Si, polymethyl methacrylate (PMMA), or octadecyltrichlorosilane (OTS)‐treated SiO_2_/Si substrates. The 3,6‐DATT single crystals were grown on bare or OTS‐treated /SiO_2_/Si Substrate by PVT (A two‐zone horizontal tube furnace under 17 Pa, and the 3,6‐DATT powder in the high‐temperature zone at 245 °C for 3 h, single crystals were obtained on the substrates in the low‐temperature zone.). Methods of crystal transfer were used to construct the devices on PMMA‐treated SiO_2_/Si substrates. The stamping electrodes method was used to construct the devices as the source/drain electrodes on an individual single crystal for charge transport investigation. Gold was used as the source and drain contacts by the stamping electrodes method. OFET characteristics were recorded by PDA FS380 and Keithley 4200 SCS in air. The mobility was extracted from the saturation region using the following equation:

(2) $$I_{\mathrm{DS}}=C_{i}\mu \left(W/2L\right){\left(V_{G}-V_{T}\right)}^{2}$$

*I*_DS_ is the drain current, *W* and *L* are the channel width and length, respectively. *C*_i_ is the capacitance of the insulation layer, and *V*_G_ and *V*_T_ are the gate voltage and threshold voltage, respectively.

### Synthesis: 3,6‐Di(Anthracen‐2‐yl)Thieno[3,2‐b]Thiophene(3,6‐DATT)

A mixture of the 3,6‐dibromothieno[3,2‐b]thiophene (0.60 g, 2 mmol), anthracen‐2‐ylboronic acid (0.98 g, 4.4 mmol), tetrakis(triphenylposphine)palladium(0) (0.14 g, 0.12 mmol), and K_2_CO_3_ (1.10 g, 8 mmol) were dissolved in toluene, ethane, and deionized water (*V*:*V*:*V* = 8:2:2 ml) and heating reflux under nitrogen for 12 h. The crude solid products were received by filtration. The slight yellow solid powder was purified two times by sublimation for device fabrication. Yield:44.5%. ^1^H NMR (600 MHz, Cl_2_CDCDCl_2_, 120°C) *δ* 8.63 (s, 2H), 8.54 (d, *J* = 9.7 Hz, 4H), 8.22 (d, *J* = 8.5 Hz, 2H), 8.13 (s, 4H), 8.01 (d, *J* = 8.9 Hz, 2H), 7.91 (s, 2H), 7.59 (s, 4H). ^13^C NMR (150 MHz) *δ* 125.67, 130.12, 132.35, 139.23. MS (ESI) m/z: 492.476. Anal. calculated for C_34_H_20_S_2_ (%): C: 82.89%, H: 4.09%, S: 13.02%. Found: C: 83.06%, H: 4.05%, S: 13.12%.

CCDC 2121604, 2125256 contains the supplementary crystallographic data for this paper. These data can be obtained free of charge from the Cambridge Crystallographic Data Centre via www.ccdc.cam.ac.uk/data_request/cif.

## Conflict of Interest

The authors declare no conflict of interest.

## Supporting information

Supporting Information

## Data Availability

The data that support the findings of this study are available from the corresponding author upon reasonable request.
