# Supplementary material for: Organic Phase‐Change Memory Transistor Based on an Organic Semiconductor with Reversible Molecular Conformation Transition
Source: Adv Sci (Weinh). 2022 Dec 3;10(4):2205694. doi: 10.1002/advs.202205694 (PMC9896068; doi:10.1002/advs.202205694)
Supplement: Supplementary file 1 — Supporting Information [file ADVS-10-2205694-s001.pdf]

ADVANCED SCIENCE
Open Access

Supporting Information

for *Adv. Sci.*, DOI 10.1002/advs.202205694

Organic Phase-Change Memory Transistor Based on an Organic Semiconductor with Reversible Molecular Conformation Transition

Yongxu Hu, Lei Zheng, Jie Li, Yinan Huang, Zhongwu Wang, Xueying Lu, Li Yu, Shuguang Wang, Yajing Sun, Shuaishuai Ding, Deyang Ji, Yong Lei, Xiaosong Chen*, Liqiang Li* and Wenping Hu*

((Supporting Information can be included here using this template))

Supporting Information

Organic phase change memory transistor Based on an Organic Semiconductor with Reversible Molecular Conformation Transition

Yongxu Hu, Lei Zheng, Jie Li, Yinan Huang, Zhongwu Wang, Xueying Lu, Li Yu, Shuguang Wang, Yajing Sun, Shuaishuai Ding, Deyang Ji, Yong Lei, Xiaosong Chen*, Liqiang Li* and Wenping Hu*

Contents

Section 1 | ^{1}H NMR and ^{13}C NMR spectra of 3,6-DATT.

Scheme S1| Synthetic routes and molecular structure of 3,6-DATT.

Figure S1 | Thermal gravimetric analysis of 3,6-DATT.

Figure S2 | Crosspolarized optical micrographs (scale bar:10 μm) and VI: fluorescence micrographs (UV light) of the 3,6-DATT crystal.

Figure S3 | (a) Normalized UV-vis spectra and fluorescence spectra of 3,6-DATT solution (10^{-5} M, THF) and crystal. (b) Fluorescence lifetime (τ) of 3,6-DATT in THF solution and single crystals.

Figure S4 | In situ micro-Raman of a 3,6-DATT single crystal during the operation cycle. Pristine: before operation; Program: V_G=-21 V; Erase: 365nm UV illumination.

Figure S5 | Variable-temperature (VT) ^{1}H NMR spectra (aromatic region) of 3,6-DATT.

Figure S6 | (a) Schematic diagram of QM/MM model. The higher-level molecule is displayed with the 'ball and stick' style and the serial numbers of atoms are shown. HOMO/LUMO distribution as well as the energy level of 3,6 -DATT at (b) RT Phase and (c) HT Phase.

Figure S7 | Huang–Rhys factor Sj versus mode frequency for (a) RT Phase and (b) HT Phase.

Figure S8 | Reorganization energy λ of 3,6 -DATT (a) RT Phase and (b) HT Phase.

Figure S9 | Transfer curves of the pristine device, after the programing of V_G = −90 V for 3 s and after the erasing of UV illumination for 3 s for 3,6-DATT field-effect transistors on the bare SiO_2/Si substrate.

Figure S10 | Transfer curves of 2,5-DAN with the same device construction method for OMT test, tested in the following order: (i) pristine (dark), (ii) program (V_G =−90 V for 15 s under darkness), (iii) erase (ultraviolet irradiation for 15 s).

Figure S11 | The cyclability of the organic phase change memory transistor.

Figure S12 | Transfer curves of the pristine device, the programmed device (V_G = −90 V for 3 s), and the "erased" device (V_G = 90 V for different times).

Figure S13 | Transfer curves of 3,6-DATT single crystal OFET with HT phase under 365 nm UV light irradiation for different times.

Table S1 | Crystal data and structure refinement for 3,6 -DATT (RT Phase) (CCDC:2121604).

Table S2 | Crystal data and structure refinement for 3,6 -DATT (HT Phase) (CCDC:2125256).

Table S3 | The dihedral angle, transfer integral and reorganization energy at different phases. The numbers of atoms are shown in Figure S6a.

Section S1. ^{1}H NMR and ^{13}C NMR spectra of 3,6-DATT.

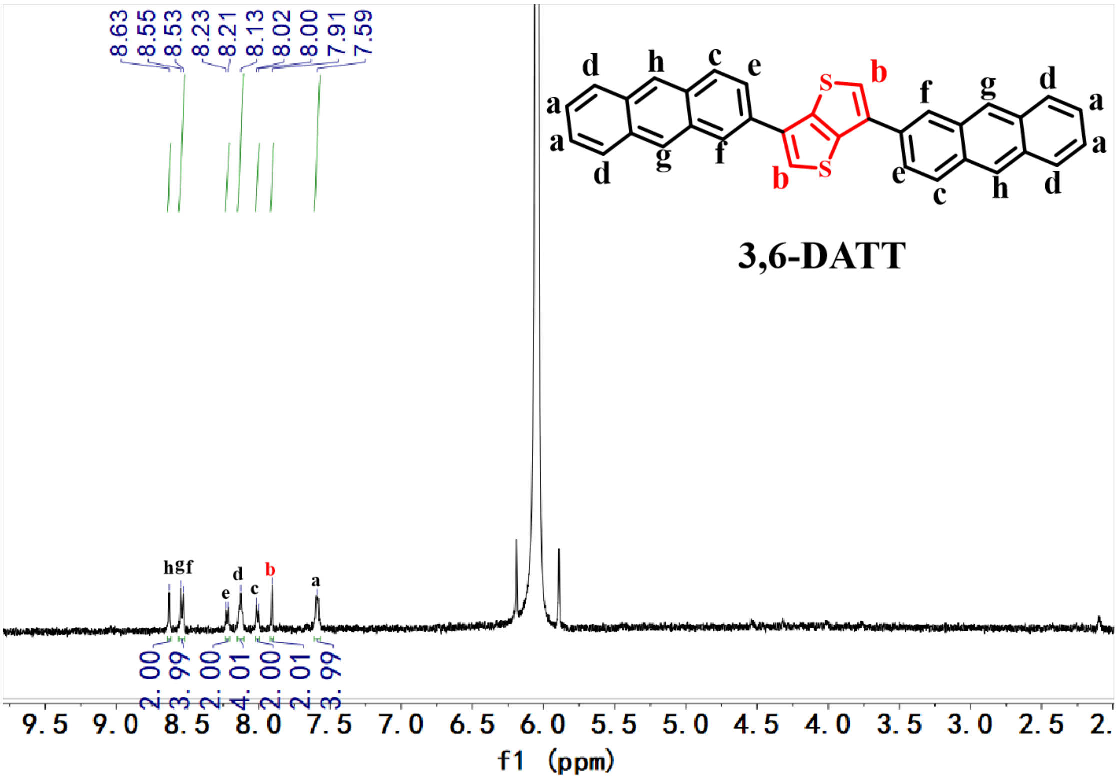

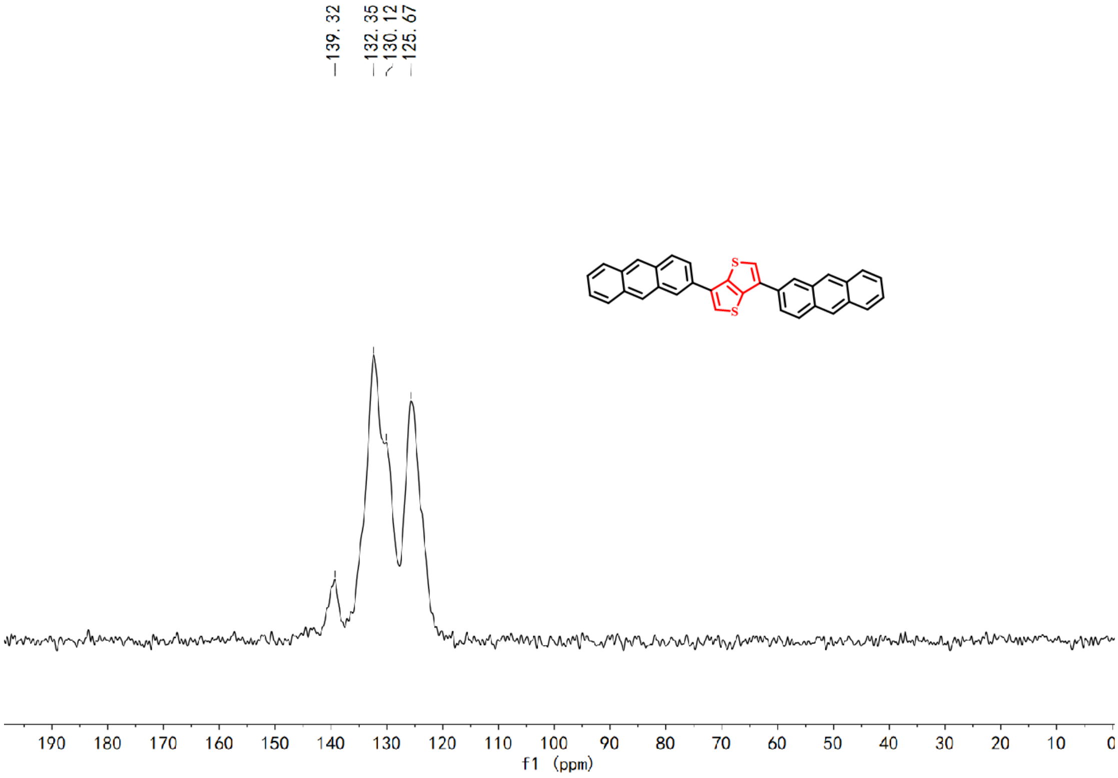

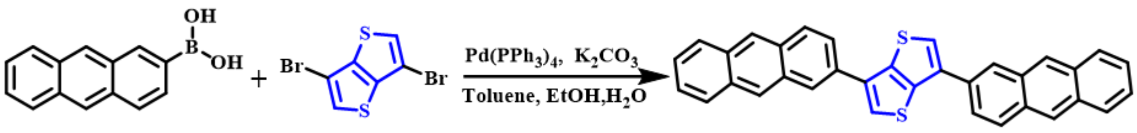

Scheme 1. Synthetic routes and molecular structure of 3,6-DATT.

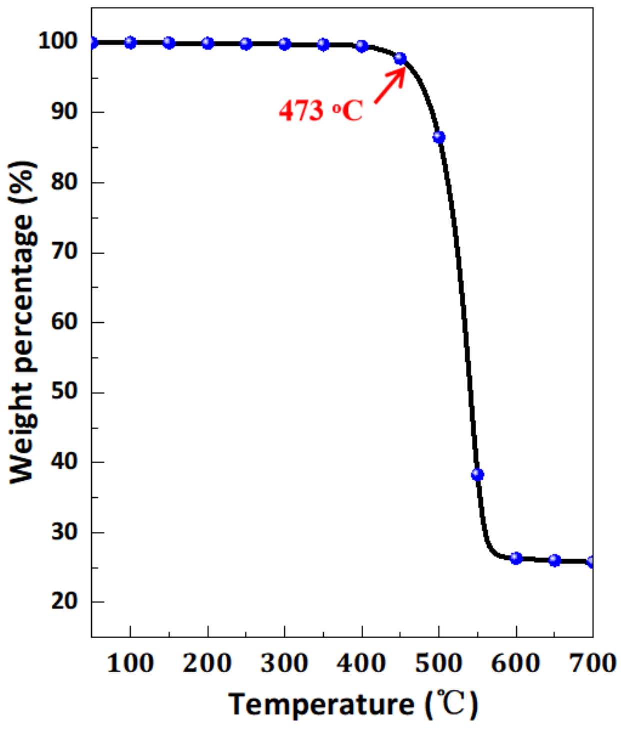

Figure S1 | Thermal gravimetric analysis of 3,6-DATT.

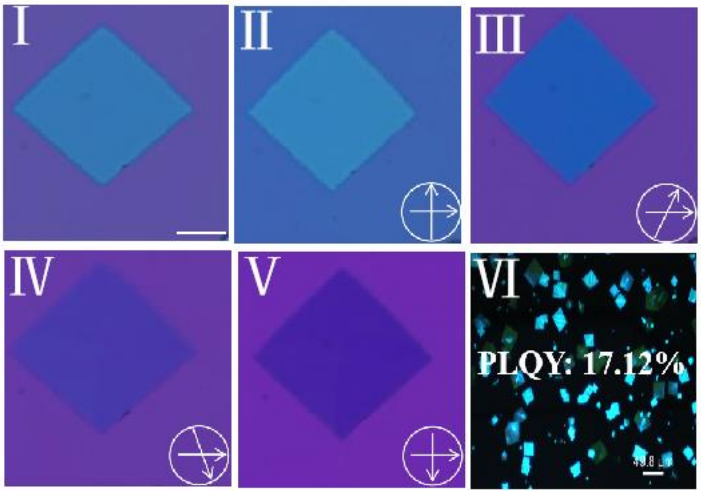

Figure S2 | Crosspolarized optical micrographs (scale bar:10 μm) and VI: fluorescence micrographs (UV light) of the 3,6-DATT crystals.

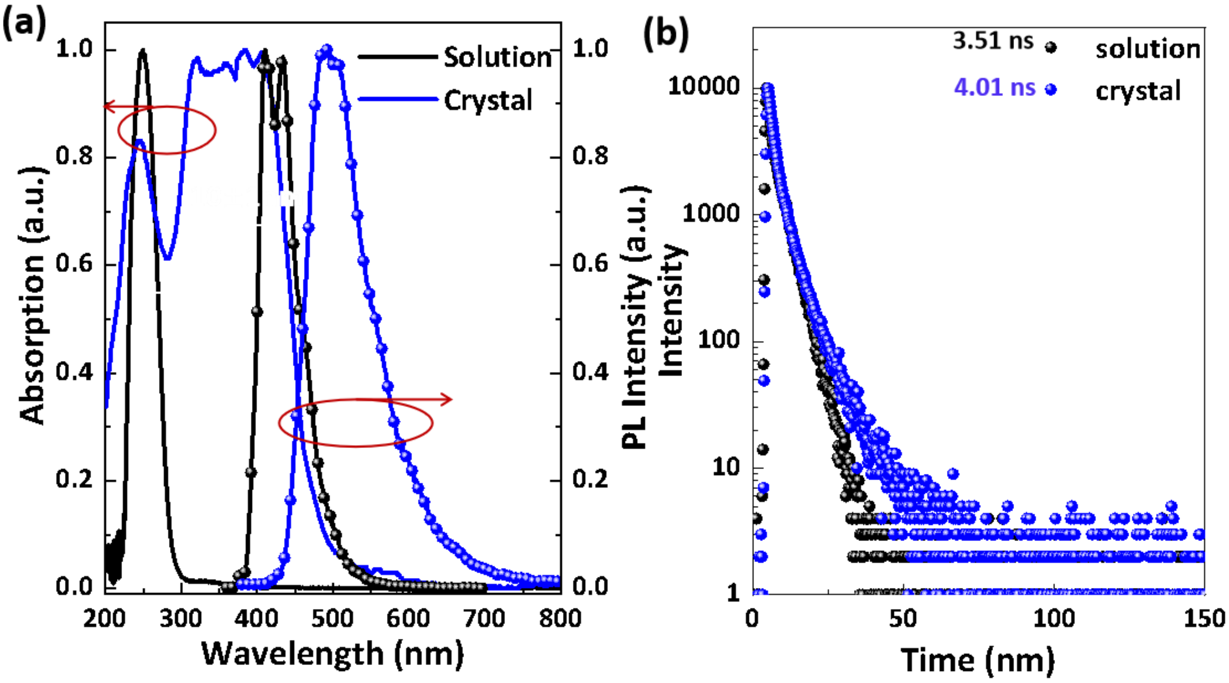

Figure S3 | (a) Normalized UV-vis spectra and fluorescence spectra of 3,6-DATT solution (10^{-5} M, THF) and crystal. (b) Fluorescence lifetime (τ) of 3,6-DATT in THF solution and single crystals.

To acquire the aggregation states information of the 3,6-DATT crystal, the UV–vis absorption and photoluminescent (PL) spectra of 3,6-DATT were taken in the solution state (in THF) and crystal state, respectively (Figure S3a). Absorption peaks at 405.2, 384.1, 314.4, and 243.8 nm were observed in the crystal, and the 0-0 fluorescence peak appeared at 489.7 nm, generating a stokes shift of 5593.9 cm^{-1}. Furthermore, PL spectra of 3,6-DATT exhibited a red-shift of ca. 56 nm in the crystal state compared with that in the solution state, indicating strong π-π interaction in the crystal state. Bright sky-blue emission can be observed under ultraviolet light irradiation (Figure S2) and the photoluminescence quantum yield value of 3,6-DATT crystalline powder was 17.12 %.

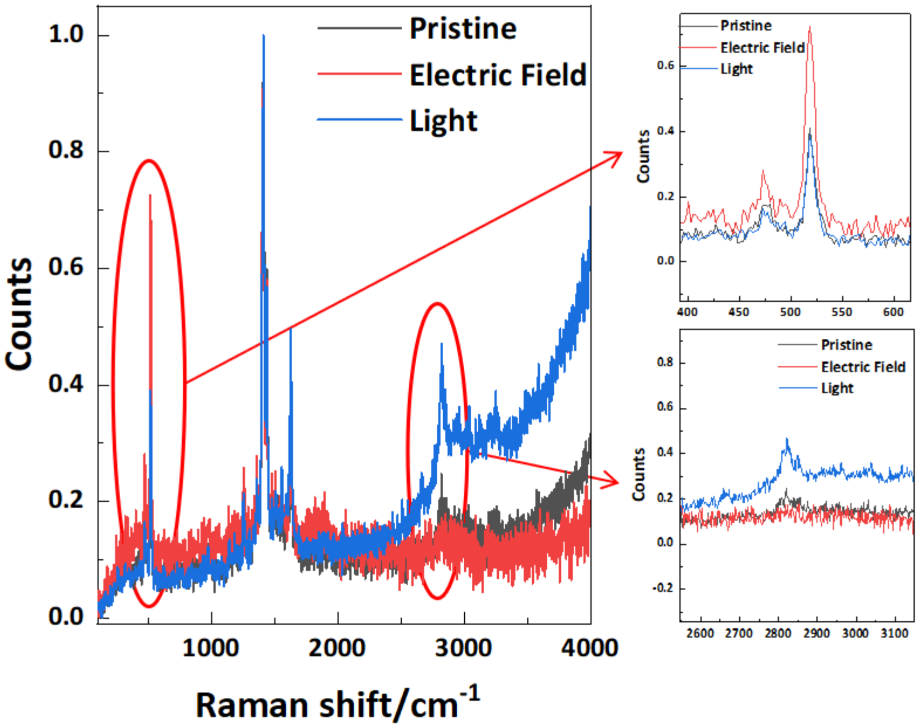

Figure S4 | In situ micro-Raman of a 3,6-DATT single crystal during the operation cycle. Pristine: before operation; Program: V_G=-21 V; Erase: 365nm UV illumination. We have detected significant changes in the C-H aromatic stretching vibrations in the 2813 cm^{-1}, and the peak center of the strongest 1432 and 1406 cm^{-1} bands was almost unchanged.

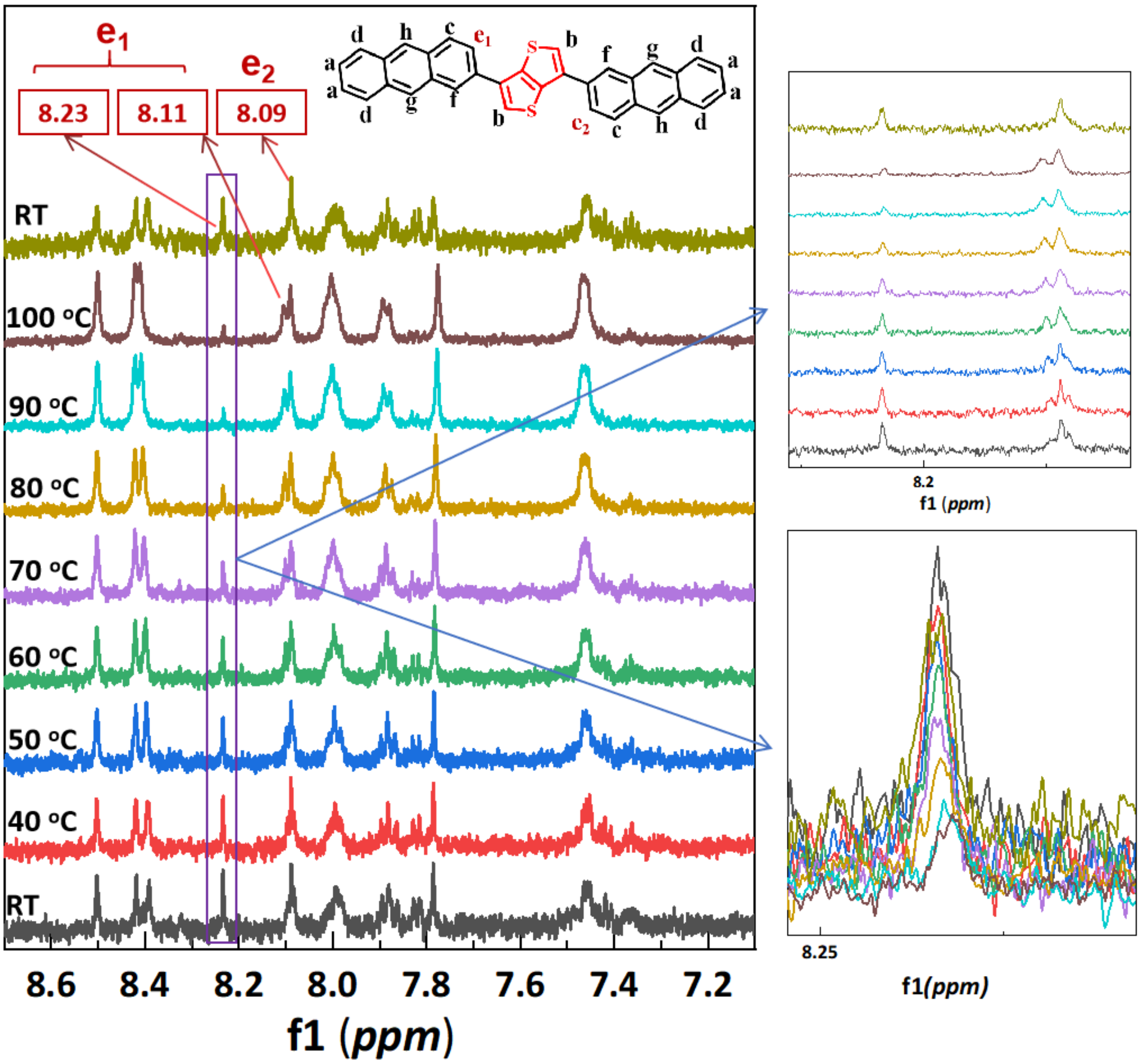

Figure S5 | Variable-temperature (VT) ^{1}H NMR spectra (aromatic region) of 3,6-DATT.

In this experiment, with the increase in temperature, the single peak chemical shift at 8.23 ppm decreased and eventually disappeared, while the double peak intensity at 8.09 ppm and 8.11 ppm increased. This change showed that the H atom at position e_1 of the benzene ring was less affected by the external magnetic field loop current, and the shielding effect was strengthened during the heating process. Therefore, the chemical shift of e_1 moved to the high field until it disappeared. This result can be proved in X-ray crystallographic analysis, the torsion angle between anthracene and thieno[3,2-*b*]thiophene at one side increased from 37.83^{o} for the RT phase to 40.98^{o} for the HT phase, and S···H bond distance became from

2.899 Å for the RT phase to 2.906 Å for the HT phase, resulting in the change of spatial configuration. The above processes can be repeated many times at room temperature and higher temperatures.

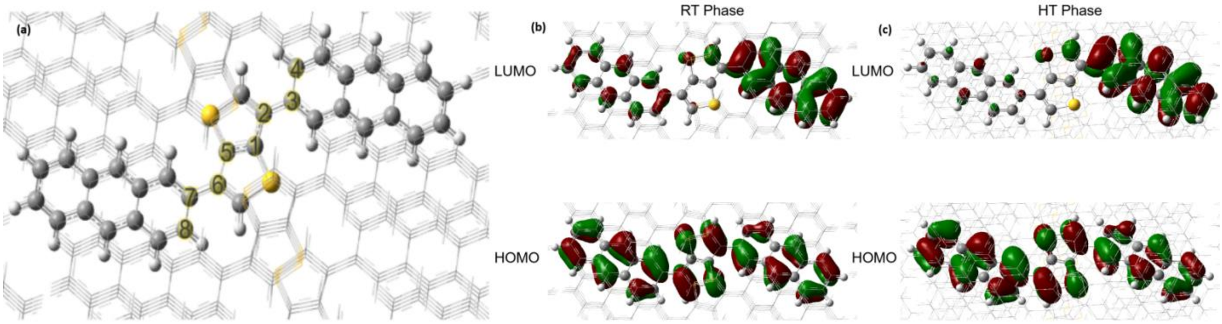

Figure S6 | (a) Schematic diagram of QM/MM model. The higher-level molecule is displayed with the 'ball and stick' style and the serial numbers of atoms are shown. HOMO/LUMO distribution as well as the energy level of 3,6 -DATT at (b) RT Phase and (c) HT Phase.

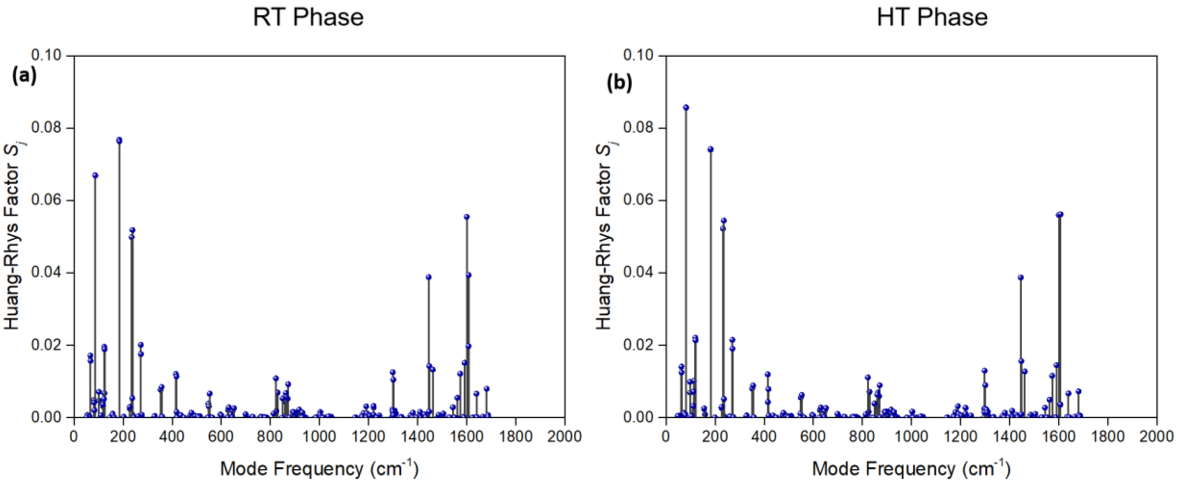

Figure S7 | Huang–Rhys factor Sj versus mode frequency for (a) RT Phase and (b) HT Phase.

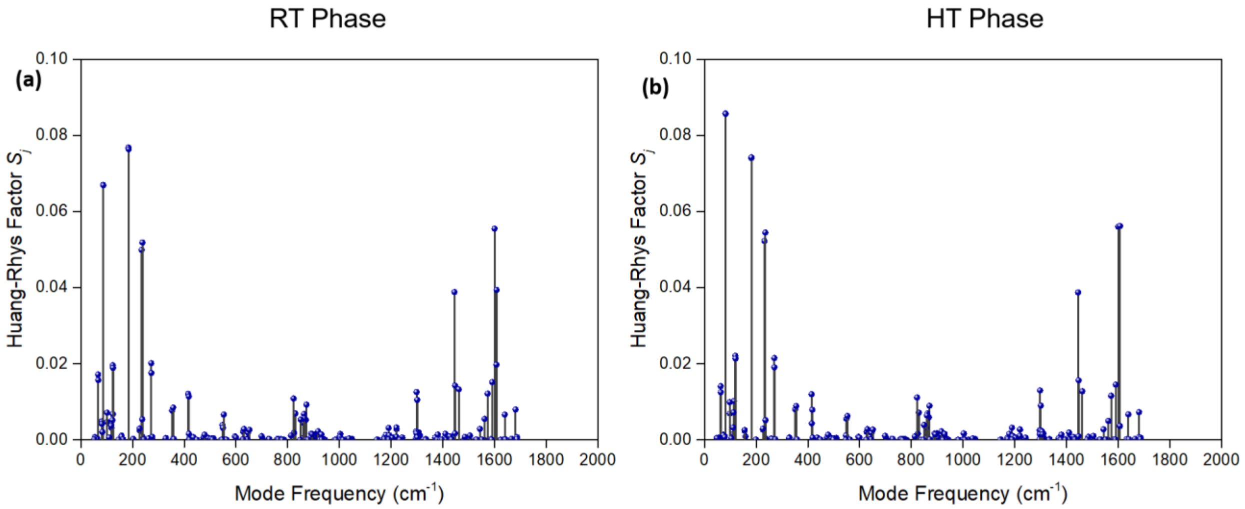

Figure S8 | Reorganization energy λ of 3,6 -DATT (a) RT Phase and (b) HT Phase.

To deeply understand the above-mentioned phenomenon, density functional theory (DFT) calculations were performed in Gaussian 09 package.[1] The optimized structures in aggregates were obtained based on two-layer quantum mechanics/molecular mechanics (QM/MM) model with an electronic embedding scheme (Figure S6a). One central 3,6-DATT molecule was set as a higher layer, calculated by B3LYP functional with 6-31G(d, p) basis set. The Grimme's dispersion corrections (D3) together with Becke and Johnson (BJ) damping were applied. The sufficient surrounding molecules were set as the lower layer, treated by UFF molecular mechanics method. No striking geometric structure variation was found, except for a little more planarization at room temperature (Table S3). The nearly temperature-independent highest occupied molecular orbital (HOMO) was delocalized over the whole molecule. While, the asymmetrically-distributed lowest unoccupied molecular orbital (LUMO) was slightly more stable at higher temperatures (Figure S6b,c).

To understand the possible mechanism behind this, the transfer integral and reorganization energy should be investigated. As one crucial factor in mobility, transfer integral reflects the strength of electron coupling. Large transfer integral commonly means superior carrier

mobility, due to the appreciable orbital overlap. While the reorganization energy, as the other important factor, indicates the strength of electron-phonon coupling, usually inversely related to the transport rate. Here, the transfer integral was calculated by PW91PW91/def2-tzvp, a reliable method widely used by previous works. There are six charge transport channels in the nearest molecular pairs, Figure 4c,4d. The fatal reduction from 15.29 meV in the RT phase to 5.26 meV in the HT phase along the P3(6) channel should be the mean reason for the mobility degradation, Table S3. On the other hand, the reorganization energy showed indistinguishable variation with temperature, in line with the similarity in geometric structure, as shown in Figure S8. The mobility as well as the corresponding parameters were calculated by MOMAP (Molecular Materials Property Prediction Package).

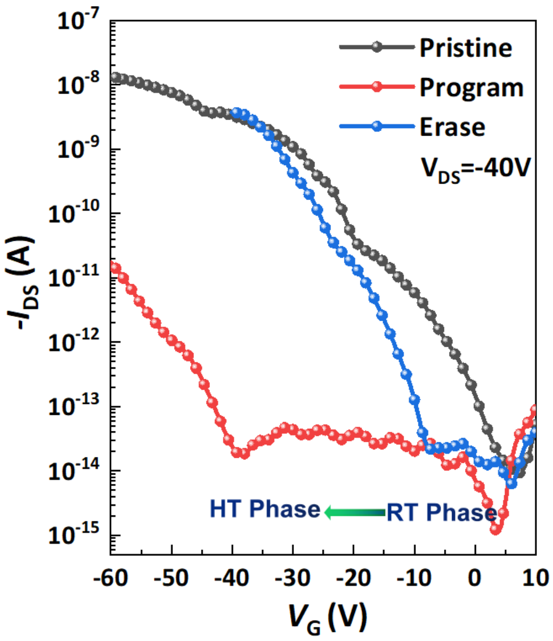

Figure S9 | Transfer curves of the pristine device, after the programming of $V_G = -90$ V for 3 s and after the erasing of UV illumination for 3 s for 3,6-DATT field-effect transistors on the bare SiO_2/Si substrate.

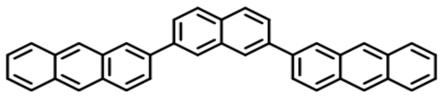

2,5-DAN

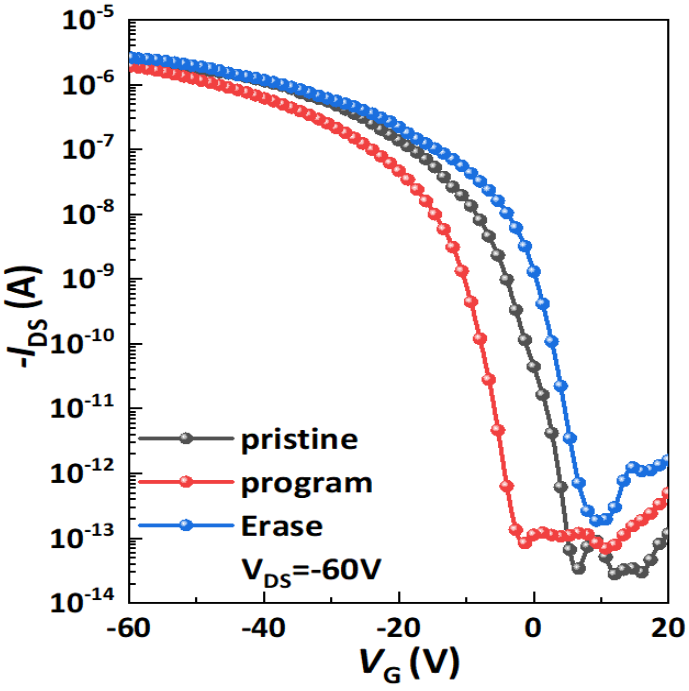

Figure S10 | Transfer curves of 2,5-DAN with the same device construction method for OMT test, tested in the following order: (i) pristine (dark), (ii) program (V_G =−90 V for 15 s under darkness), (iii) erase (ultraviolet irradiation for 15 s).

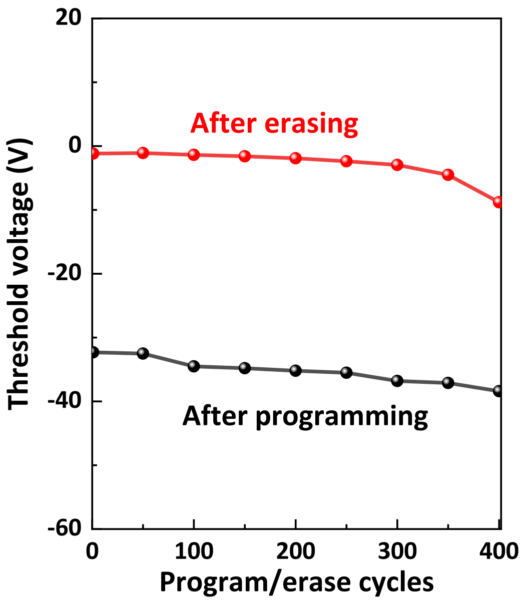

Figure S11 | The cyclability of the organic phase change memory transistor.

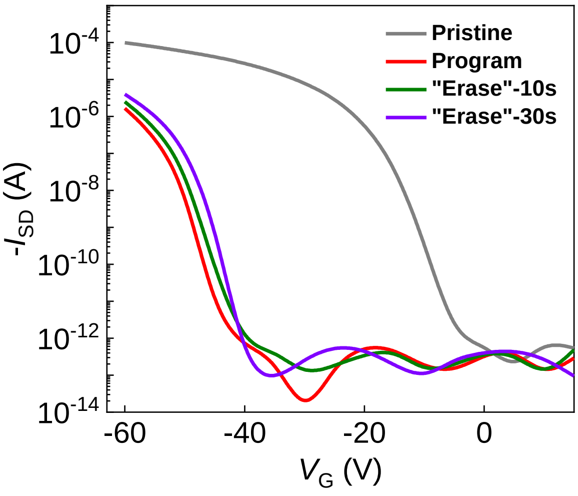

Figure S12 | Transfer curves of the pristine device, the programmed device ($V_G = -90$ V for 3 s), and the "erased" device ($V_G = 90$ V for different times).

The transition from the HT phase to the RT phase will not occur under positive gate voltage. As shown in Figure S12, the device with HT phase was applied with positive gate voltage for different times, but the transfer curves only display a slight change, which is not enough to return to the pristine state.

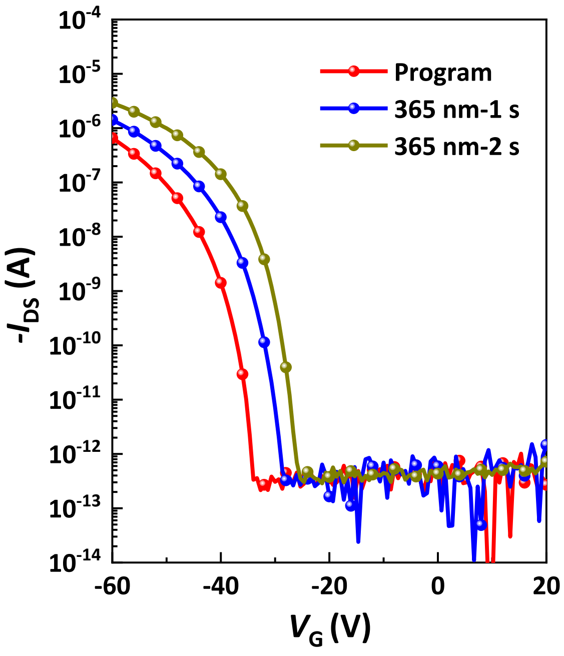

Figure S13 | Transfer curves of 3,6-DATT single crystal OFET with HT phase under 365 nm UV light irradiation for different times.

To exclude the effect of photoelectric response on the erasing operation, we measured the transfer characteristics of OFET based on HT phase crystal under light irradiation for different times. The transfer curves of OFET based on HT phase crystal were shown in Figure S13 under UV light irradiation for 1 s and 2 s. Other experimental conditions are the same as the erasing operation in the maintext. The photoelectric response of organic semiconductors under UV light irradiation is a relatively fast process (<1 s)[2] compared to molecular conformation transition (3 s in this case). The irradiation time in this reference experiment should be long enough to complete the photoelectric response. However, the transfer curves of the device under UV light irradiation for short time (1 s and 2 s) are far away from the pristine state of the RT phase crystal. Therefore, it is deduced that the photoelectric response effect only plays a minor role in the erasing operation.

In addition, combined with the results of the Raman spectrum, PL spectrum, XRD patterns, and ^{1}H NMR spectra, it is believed that the molecular conformation transition plays a dominant role in the erasing operation.

Table S1 | Crystal data and structure refinement for 3,6 -DATT (RT Phase) (CCDC:2121604).

Compounds		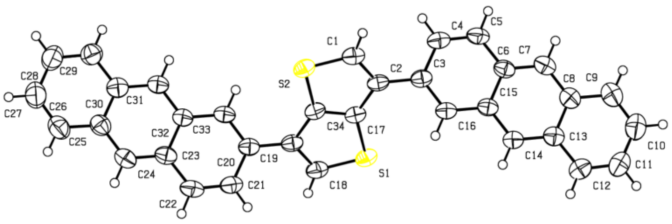
Empirical Formula		$C_{34}H_{20}S_2$
M_r		492.62
Temperature/K		303 K
Space group		P 1 21/n 1
Unit Cell Lengths (Å)	a	7.1574 (2)
	b	7.3756 (2)
	c	44.3079 (13)
Unit Cell angles (°)	α	90
	β	93.013 (2)
	γ	90

Cell Volume (Å^3)	2335.79 (11)
Z	4
ρ_{calc} g/cm^3	1.401
Mu/mm^{-1}	2.228
F(000)	1024.0

Table S2 | Crystal data and structure refinement for 3,6 -DATT (HT Phase) (CCDC:2125256).

Compounds		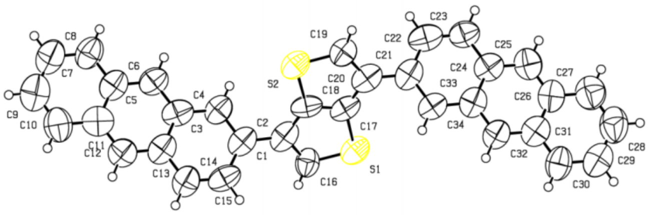
Empirical Formula		$C_{34}H_{20}S_2$
M_r		492.62
Temperature/K		395 K
Space group		P 1 21/n 1
Unit Cell Lengths (Å)	a	7.1435 (3)
	b	7.4589 (3)
	c	44.502 (3)
Unit Cell angles (°)	α	90
	β	92.285(2)
	γ	90
Cell Volume (Å^3)		2369.3 (2)
Z		4
ρ_{calc} g/cm^3		1.381
Mu/mm^{-1}		2.196
F(000)		1024.0

Table S3 | The dihedral angle, transfer integral and reorganization energy at different phase. The numbers of atoms are shown in Figure S6a.

	∠C1C2C3C4 (°)	∠C5C6C7C8 (°)	Transfer integral (meV)			Reorganization energy (meV)
			P1(4)	P2(5)	P3(6)	
RT Phase	143.35	142.55	11.19	2.56	15.29	97.2
HT Phase	142.57	141.87	11.47	4.06	5.26	97.1

[1] M. Frisch, G. Trucks, H. Schlegel, G. Scuseria, M. Robb, J. Cheeseman, G. Scalmani, V. Barone, G. Petersson, H. Nakatsuji, X. Li, M. Caricato, A. Marenich, J. Bloino, B. Janesko, R. Gomperts, B. Mennucci, H. Hratchian, J. Ortiz, A. Izmaylov, J. Sonnenberg, Williams, F. Ding, F. Lipparini, F. Egidi, J. Goings, B. Peng, A. Petrone, T. Henderson, D. Ranasinghe, V. Zakrzewski, J. Gao, N. Rega, G. Zheng, W. Liang, M. Hada, M. Ehara, K. Toyota, R. Fukuda, J. Hasegawa, M. Ishida, T. Nakajima, Y. Honda, O. Kitao, H. Nakai, T. Vreven, K. Throssell, J. Montgomery, J. Peralta, F. Ogliaro, M. Bearpark, J. Heyd, E. Brothers, K. Kudin, V. Staroverov, T. Keith, R. Kobayashi, J. Normand, K. Raghavachari, A. Rendell, J. Burant, S. Iyengar, J. Tomasi, M. Cossi, J. Millam, M. Klene, C. Adamo, R. Cammi, J. Ochterski, R. Martin, K. Morokuma, O. Farkas, B. Foresman, Fox, D. J. *Gaussian 09 Rev. E.01*, Wallingford, CT, 2013.

[2] K.-J. Baeg, M. B., D. Natali, M. Caironi, Y.-Y. Noh, *Adv. Mater.* **2013**, *25*, 4267.
